# The Bridge2AI-voice application: initial feasibility study of voice data acquisition through mobile health

**DOI:** 10.3389/fdgth.2025.1514971

**Published:** 2025-04-15

**Authors:** Elijah Moothedan, Micah Boyer, Stephanie Watts, Yassmeen Abdel-Aty, Satrajit Ghosh, Anaïs Rameau, Alexandros Sigaras, Olivier Elemento, Bridge2AI-Voice Consortium, Yael Bensoussan, Yael Bensoussan

**Affiliations:** ^1^ Charles E. Schmidt College of Medicine, Florida Atlantic University, Boca Raton, FL, United States; ^2^ USF Health Voice Center, Department of Otolaryngology-Head & Neck Surgery, University of South Florida, Tampa, FL, United States; ^3^ McGovern Institute for Brain Research, Massachusetts Institute of Technology, Cambridge, MA, United States; ^4^ Sean Parker Institute for the Voice, Department of Otolaryngology-Head & Neck Surgery, Weill Cornell Medical College, New York, NY, United States; ^5^ Englander Institute for Precision Medicine, Weil Cornell Medical College, New York, NY, United States

**Keywords:** artificial intelligence, voice, biomarkers, mobile application, voice biomarkers

## Abstract

**Introduction:**

Bridge2AI-Voice, a collaborative multi-institutional consortium, aims to generate a large-scale, ethically sourced voice, speech, and cough database linked to health metadata in order to support AI-driven research. A novel smartphone application, the Bridge2AI-Voice app, was created to collect standardized recordings of acoustic tasks, validated patient questionnaires, and validated patient reported outcomes. Before broad data collection, a feasibility study was undertaken to assess the viability of the app in a clinical setting through task performance metrics and participant feedback.

**Materials & methods:**

Participants were recruited from a tertiary academic voice center. Participants were instructed to complete a series of tasks through the application on an iPad. The Plan-Do-Study-Act model for quality improvement was implemented. Data collected included demographics and task metrics including time of completion, successful task/recording completion, and need for assistance. Participant feedback was measured by a qualitative interview adapted from the Mobile App Rating Scale.

**Results:**

Forty-seven participants were enrolled (61% female, 92% reported primary language of English, mean age of 58.3 years). All owned smart devices, with 49% using mobile health apps. Overall task completion rate was 68%, with acoustic tasks successfully recorded in 41% of cases. Participants requested assistance in 41% of successfully completed tasks, with challenges mainly related to design and instruction understandability. Interview responses reflected favorable perception of voice-screening apps and their features.

**Conclusion:**

Findings suggest that the Bridge2AI-Voice application is a promising tool for voice data acquisition in a clinical setting. However, development of improved User Interface/User Experience and broader, diverse feasibility studies are needed for a usable tool.

**Level of evidence**: 3.

## Introduction

The human voice constitutes a rich source of information as it relates to disease status (1). With its spectrum of acoustic features coupled with its cost-effectiveness and accessibility, voice has gained recognition for its utility as a potential biomarker for disease, screening, diagnosis and monitoring (2, 3). Furthermore, recent development technology, such as artificial intelligence (AI) and machine learning (ML), have seen its introduction into the realm of voice analysis that can now be automated to process large amounts of data (1). Combining AI/ML with voice analysis allows for efficient analysis of voice data, which promises discovery of scalable acoustic markers in association with health diagnosis, screening, and monitoring to improve patient outcomes (4).

Voice data collection is low cost inexpensive, often only requiring recording device with a microphone (i.e., computer, smart device). This simplicity makes voice-based screening and diagnostics an attractive tool to utilize in low-resource settings. However, to unlock the full potential of voice as a tool, there is a crucial need for large datasets that capture diverse populations and disease statuses along with other established physiologic biomarkers (2, 5, 6). Current literature on this topic has only been studied on small- to medium-sized data sets with limited data outside of acoustic measures not linked to multi-modal health data. Inclusion of speech and voice data in large-scale trials adds an additional longitudinal variable that has the potential to improve scientific discovery and patient outcomes (7), but comparing studies and pooling data is challenging due to a lack of existing standards in how voice and other acoustics are measured and collected (8).

In hopes of advancing the potential of voice as a biomarker, the Bridge2AI-Voice consortium has the goal of establishing an ethically sourced, diverse, and publicly available voice database linked to multimodal health biomarkers (9). This extensive and open-access voice database will serve as the foundation for voice AI research, facilitating the development of predictive models that can significantly advance the field of voice through improved quality acoustic data, establishment of voice bioinformatic standards, development of an infrastructure for audiomic data storage, and formulation of training algorithms for clinicians and scientists.

In order to create this voice database, the Bridge2AI-Voice Consortium developed a novel mobile application hosting the data acquisition protocols to collect data through various acoustic tasks, surveys, questionnaires, and validated patient-reported outcomes (PROs). With the goal of creating data collection with users at home, there needs to be an evaluation of its utility in order to identify technical constraints and challenges that exist. A pilot feasibility study allows for us to gain a preliminary understanding of user interaction and general feedback of this app for a smoother transition to broad implementation (10). This pilot feasibility study assesses the possible implementation of this application through task performance metrics and participant feedback.

## Materials & methods

### Study setting and participants

This study was conducted at a tertiary academic voice center, University of South Florida Health Voice Center, in Tampa, Florida between June 5, 2023, and July 28, 2023. The study used a mixed sample of participants, with and without voice disorders. The eligibility criteria included: participants who were at least 18 years old and could read the English language. Exclusion criteria were as follows: an inability to provide informed consent in English and inability to read English. All patients meeting inclusion criteria were offered to participate in the study.

### Enrollment

Participants were recruited by providers or research staff for enrollment. Participants were informed during the consent process that the app was created by the Bridge2AI-Voice consortium and outlined its purpose. Participants were explicitly informed about the data that was being collected, the methods used to secure these data, and the information that would be used for the study. All participants provided written informed consent for all the study procedures. The participants did not receive financial incentives for completion of the study. The study was approved by the Institutional Review Board of the University of South Florida (IRB number 004890).

### App development

A multi-institutional, multi-disciplinary group of researchers participated in the development of this novel tool. The group consisted of researchers from 14 different institutions and with expertise in software engineering, data science, machine learning, laryngology, speech pathology, acoustic science, bioethics, pulmonary science, neurological biomarkers, and mood biomarkers. The aim of the app was to collect demographic information, validated questionnaires, and acoustic tasks for four different categories of diseases in the adult population: vocal pathologies, neurological and neurodegenerative disorders, mood and psychiatric disorders, and respiratory disorders. Full protocols for data acquisition were developed including the following categories:

- Demographics: The group was asked to include common demographic data and include other demographics that could affect voice and speech (e.g., weight, socio-economic status, literacy status, etc.).
- Past medical history (PMHx): The group was asked to include common disorders with care being taken to include diseases and conditions that are known to affect voice and speech (e.g., COPD, chronic sinusitis).
- Confounders: The group was asked to include confounders and social habits that are known to affect voice and speech (e.g., smoking status, hydration status).
- Acoustic tasks: The group was asked to include common acoustic tasks performed for screening or diagnosis of the conditions studied in the clinical setting or research setting.
- Validated questionnaires and PROs: Patient-reported outcomes and validated patient questionnaires commonly used in clinical or research practice with evidenced-based correlation with the diseases studied (e.g., GAD-7 for anxiety, VHI-10 for dysphonia).
- Clinical Validation: The group was asked to develop a section including questions that would confirm the diagnosis and treatment obtained by a clinician.
- “Gold Standards”: The group was asked to add data modality that are used for confirmation or included in the basic work-up of the diseases studied (e.g., pathology report for laryngeal cancer, pulmonary function test for asthma).

Full data acquisition protocols will be available in the REDCap instrument Shared Library and are also available for download at our GitHub repository: https://github.com/eipm/bridge2ai-redcap.

**Figure 1:**
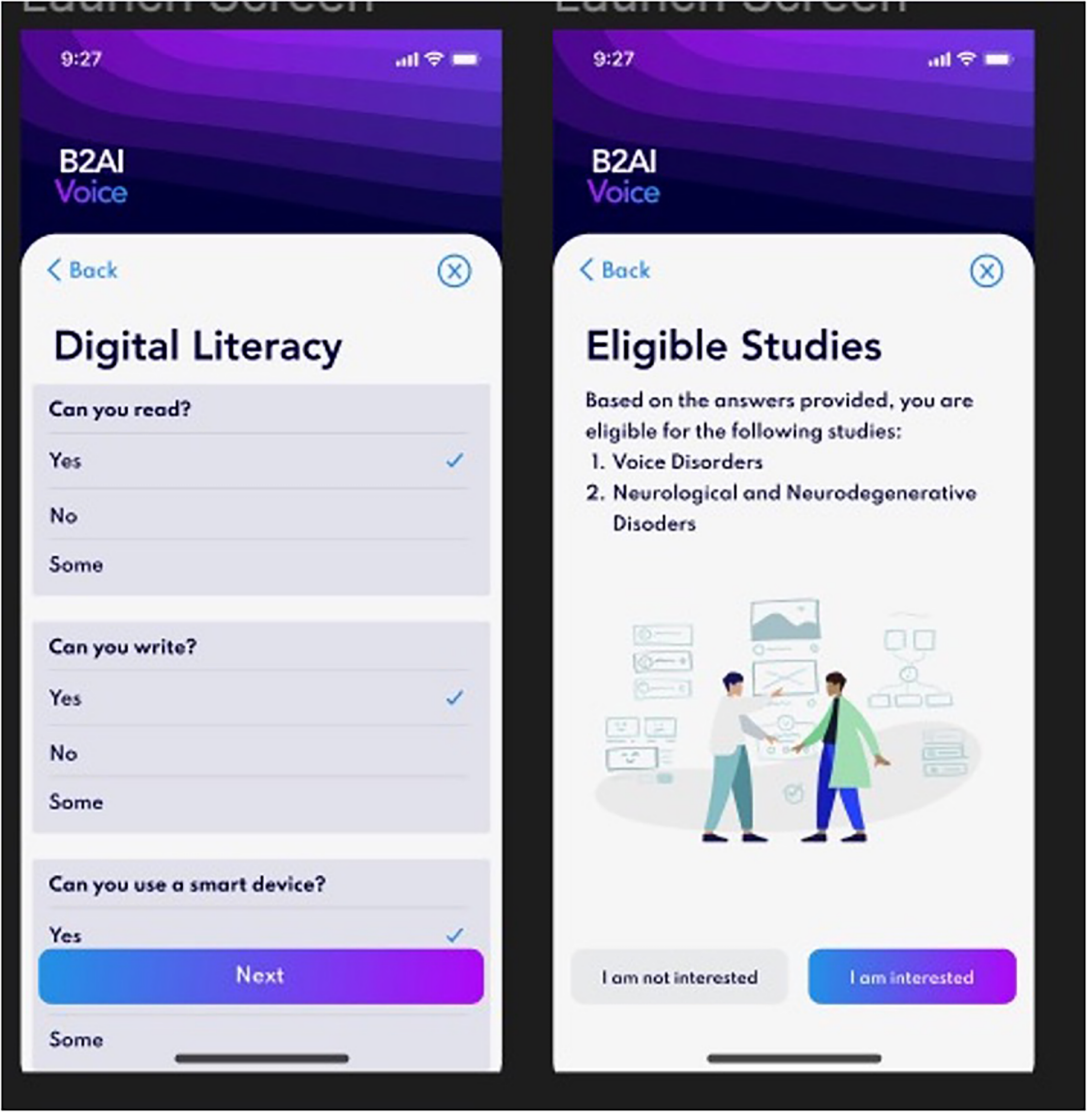
Bridge2AI-Voice app interface.

**Figure 2:**
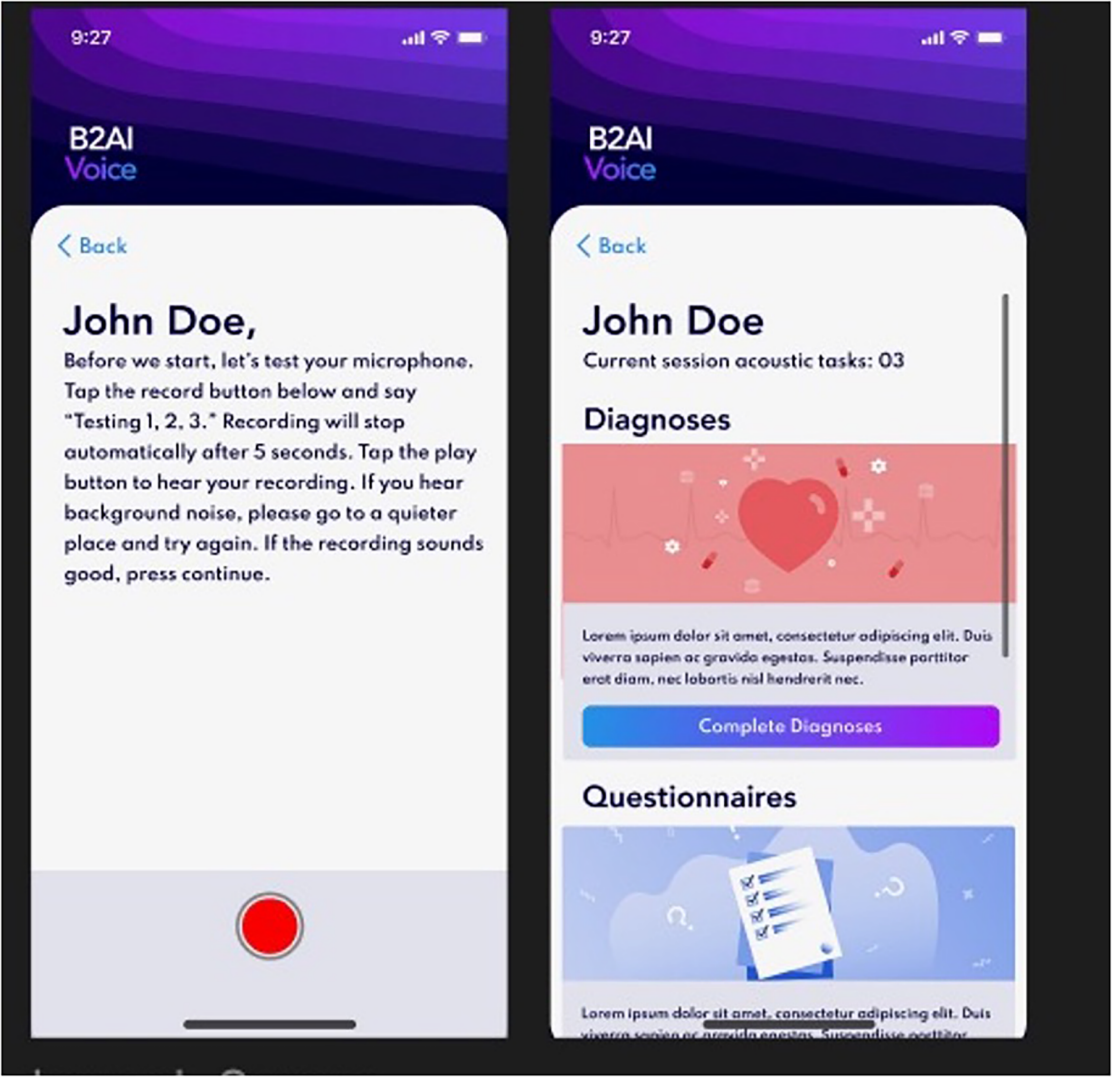
Bridge2AI-Voice app interface.

All current tasks on the app during the study period are listed in Table 1. The Bridge2AI-Voice is undergoing constant alpha- and beta-testing in order to better understand its practicality and usability among the general public before large-scale data collection and therefore, some tasks may be altered, added, or removed based on patient feedback, auditing and validation experiments conducted by our group (11). Figures 1, 2 showcase the current design of the app.

**Table 1:** Available tasks, PROs, questionnaires, and mean time for completion on the Bridge2AI-voice app.

Task	Type of task	Mean time for completion (min:sec)
Demographics	Questionnaire	2:29
Confounders	Questionnaire	11:22
Voice perception	Questionnaire	0:20
Voice problem severity	Questionnaire	0:12
Voice handicap index-10 (VHI-10)	Validated PRO	0:37
Patient health questionnaire-9 (PHQ-9)	Validated PRO	1:19
General anxiety disorder-7 (GAD-7)	Validated PRO	1:04
Positive and negative affect scale (PANAS)	Validated PRO	0:49
Custom affect scale	Validated PRO	1:14
DSM-5 adult	Validated PRO	5:24
PTSD adult	Validated PRO	2:47
ADHD adult	Validated PRO	2:23
Audio check	Acoustic Task	0:30

### Outcome measures

#### Demographics

Participants completed a basic sociodemographic questionnaire at enrollment which included: age, gender, primary language, education level, employment status. Additional information collected included any history of a voice disorder, self-reported disabilities, smart device ownership, and mobile health app use.

### Feasibility metrics

A six-item feasibility metric questionnaire was created by the research team to better understand participant feedback. Metrics related to task completion and time of completion were collected for every task; other metrics were only answered when applicable to task. Completion time did include any time that research staff was asked for assistance and assisted. Feasibility metrics were answered as yes (Y) or no (N). Answers were determined by the research staff collecting data.

• Was the task completed?
• Was the acoustic task successfully recorded?
• Did the acoustic task have to be re-recorded?
• Was a headset used?
• What was the time of completion?
• Did the participant ask for assistance?

### Exit survey

A 6-item interview-style questionnaire was given to participants at the end of the study completion to better understand participants engagement/interaction and to gauge general feedback. Exit survey questions, and subsequent follow up questions, were modified from the Mobile App Rating Scale (MARS) from the Functionality and Engagement sections (12). Responses were qualitative and not scored on a scale.

1. How easy were the tasks prompts to understand?
  • Was the vocabulary, wording, and grammar clear, unambiguous, and appropriate?
  • Did you have to go back and reread the prompt to understand was it was asking for?
2. How easy was the app to interact with?
  • Did you understand how to interact with the app to successfully complete the tasks?
  • Were the interactions consistent and intuitive?
  • Did you understand whether you had completed a task correctly, how to progress to the next screen, etc.?
3. Was the app interesting/engaging for you to use?
4. Did you find the tasks physically difficult or taxing to perform?
5. Did you find the tasks mentally difficult or taxing to perform?
6. Was the interface physically difficult to interact with (e.g., taps, swipes, pinches, scrolls)?

### Data collection

Participants were brought to a private clinic room and introduced to the app on a study iPad by a member of the research staff. Each participant was asked to complete a one to three tasks followed by the feedback interview for a total time of less than 20 min. Participants were then informed of what task(s) they would be completing, that they would be timed from when they began until they had completed the task, and that a research member would be available for assistance/questions if needed. Participants were instructed to wear a headset with microphone if the task included voice recording, as per the Bridge2AI-Voice suggested standards, and begin. A research member observed and timed the participant. At completion of each task, time to completion and feasibility metrics were recorded. The research personnel then began the exit survey questionnaire with participants in which qualitative responses were recorded. This procedure was completed for each task the participant completed. Participants were limited to 1–3 tasks at a time, in which task assignment depended on the length of the task and the time available by the participant. Audio data was not collected at this stage of the app development. Current research by the consortium is attempting to outline techniques and recommend appropriate protocols for quality voice data collection in future iterations of the app as well as other clinical research involving voice data collection (13).

### PDSA model of improvement

We employed the Plan-Do-Study-Act (PDSA) model for three phases of data collection (14). The PDSA model is a four-step iterative approach for quality improvement and is widely used in quality improvement initiatives. The model begins with a strategy to assess improvement approaches (Plan), followed by a small-scale trial of data collection (Do). The study team evaluates and gains insight from the outcomes (Study), determining whether to implement alterations or initiate a new cycle of improvement (Act). This model was used to inform the app development team of weaknesses or concerns observed by the research team during participant completion. The phases in this study consisted of 10–20 participants per cycle. Minimal yet effective improvements related to recruitment, data recording, and interface changes were made. None of the improvements made affected participant data collection and subsequent metric measurement. No major changes to features or content were made to the app during the research study timeline.

## Results

### Overview

47 participants were recruited over a two-month enrollment period. Participant characteristics are shown in Table 2. Mean and median age was 58.3 and 64, respectively. 61.7% were female, 91.5% spoke English as a primary language, 55.3% held a bachelor's or graduate degree, and 40.4% were employed. 36% of participants had a self-reported disability, most commonly reporting a physical, visual, or auditory impairment/deficit. 100% of participants owned a smart device, with 49% using a mobile health application currently. Over 20 primary referral diagnoses were reported by participants in Table 3.

**Table 2:** Participant characteristics (*n* = 47).

Characteristic	Value^b^
Age in years, median (range)	58.3 (19–92)
Gender, *n* (%)	
Male	18 (38.3%)
Female	29 (61.7%)
Highest Level of Education, *n* (%)	
High School Diploma	7 (14.9%)
Some College	8 (17%)
Associate's Degree	6 (12.8%)
Bachelor's Degree	17 (36.2%)
Graduate Degree	9 (19.1%)
Primary Language, *n* (%)	
English	43 (91.5%)
Other^a^	4 (8.5%)
Employment Status, *n* (%)	
Student	3 (6.4%)
Employed	19 (40.4%)
Retired	21 (44.7%)
Unemployed	1 (2.3%)
Disability	3 (6.4%)
Self-Reported Disability Status, *n* (%)	
Yes	17 (36.2%)
No	33 (63.8%)
Own a smartphone or tablet? *n* (%)	
Yes	47 (100%)
No	0 (0%)
Do you use a mobile health application? *n* (%)	
Yes	23 (48.9%)
No	24 (51.1%)

^a^ Other languages included Spanish, Mandarin, Bengali, and Thai.

^b^ Percentages may not sum to 100% due to rounding.

**Table 3:** Participant voice diagnoses.

Irritable Larynx Syndrome	Chronic Cough	Vocal Cord Paralysis
Vocal Cord Hypomobility	Vocal Cord Leukoplakia	Muscle Tension Dysphonia
Interstitial Lung Disease	Chronic Obstructive Pulmonary Disease	Spasmodic Dysphonia
Velopharyngeal Insufficiency	Recurrent Respiratory Papillomatosis	Sulcus
Oropharyngeal Dysphagia	Asthma	Amyloidosis
Gastroesophageal Reflux Disease	Presbyphonia	Vocal Cord Paresis
Vocal Cord Scarring	Current/Post Tracheostomy Tube	History of Glottic Cancer/High Grade Dysplasia

Three PDSA cycles were completed. There were 15 participants for PDSA 1, 20 participants for PDSA 2, 12 participants for PDSA 3. PDSA 1 focused on improving recruitment practices of participants, PDSA 2 focused on improving the research staff assistance, and PDSA 3 focused on improving feedback relaying to the app development team. Alpha- and beta-testing as well as app updates were ongoing throughout this study period.

### Feasibility metrics

There was a total of 29 different questionnaires and tasks at the time of data collection (Table 1). The “confounders” questionnaire was stratified into 5 different “tasks”, thus a total of 34 “tasks” were available to complete. The 47 participants completed a total of 68 tasks. Of these 68, only 46 (68%) were able to successfully complete the task as instructed. Moreover, of these 68 tasks, 32 fell under the “acoustic” category in which an audio recording by the participant was required. 13 (41%) were able to successfully complete as instructed. Participants asked for assistance by the research staff 41.2% of the time, often asking multiple times for an individual task. Notably, the Glides task (i.e., a task requiring moving from high to low and low to high pitches) required assistance 100% of the time. A total of 19 participants asked for assistance on one or multiple tasks with a mean age of 63.8. Those who asked for assistance were mainly female (66.6%), employed (46.7%), and held a bachelor's degree (43.3%).

Table 1 reports the average completion time for each task. When all current task average completion times were added together, the total completion time of all tasks in the app was approximately 51 min and 30 s. The longest task to complete was the DSM-5 Adult survey and the shortest task to complete was the Voice Problem Severity scale.

### Exit survey

Upon completion of the 2-month study period, 47 participants completed the interview-style questionnaire.

The user responses reflected a favorable perception of a voice-screening app and its features, with one participant saying, “I am excited for this app to be ready one day. I would definitely use something like this with my condition”.

Moreover, responses also highlighted the utility of the application as it currently stands. One user mentions that “[they] thought it was very easy and intuitive to complete”. However, a majority of users made comments in regard to the current interface and/or with the clarity of the instructions. Many users emphasized the need for more explicit instructions regarding how to audio record the task, how to play back the recorded audio, or even when to record. One user says “I couldn't remember what the scale meant, and I had to keep scrolling back up to remind myself what it meant and then scroll way back down to where I left off” in regard to the PTSD Adult survey. Beyond this, some users felt that some of the survey and questionnaire tasks on the app were dense and difficult to engage with, making some tiresome to complete. One user notes that “I felt that the questionnaire had too many questions on the screen and could have been made into two pages” in regard to the DSM-5 Adult questionnaire.

Additionally, user responses pointed out different ways to improve the app design and experience. While the app is currently in its base model, with design and aesthetic being developed, participants suggested different modalities that could potentially reduce mental exhaustion. One user suggested an incorporation of some motivational elements to better user engagement.

## Discussion

### Principal findings

The Bridge2AI-Voice consortium developed and pilot-tested a novel mobile application designed for eventual voice data collection to improve voice data research. This study aimed to assess the practicality and utility of this application through task performance metrics and participant discussion. Results highlight that the feasibility of utilizing the data collected through this app presents both promises and challenges that need to be addressed.

While completion rates did vary across tasks, the majority of users were able to successfully complete the tasks as instructed indicating a certain level of usability. However, a majority of the acoustic tasks that would require audio collection were unsuccessfully completed, which is a very important finding to consider as we eventually aim to transition data collection in the remote setting, without assistance from research personnel. With Bridge2AI-Voice's ultimate goal of introducing at-home data collection with this mobile app, this highlights a concern that needs to be addressed. If voice and audio tasks were unable to be performed, subsequently the app would be collecting insufficient voice and audio data, weakening the diversity of the database and consequently the AI/ML models to be trained. Addressing this fundamental issue needs to be *a priori*ty for the Bridge2AI-Voice consortium in order to ensure the best voice practices and its technology are being employed in order to capture of the best audio samples. Through results of this feasibility study, a special focus on user experience/user interface (UX/UI) was initiated, with the addition of a UX/UI expert to the team for further iterations of the app.

Furthermore, based on the feasibility metrics, the task completion time remains a barrier for this application to be an efficient screening tool. As it currently stands, the summed average time for completion of all tasks available on the app is 51 min and 30 s. It's important to highlight this is subject to change as the app continues to be updated and modified, but if more elements are added to the protocol, it is reasonable to assume that this total completion time is to increase. However, one goal of the app is to ideally bundle tasks and surveys when appropriate in relation to a user's disease status. Regardless, this raises concern about user fatigue and engagement sustainability. This fatigue experienced often towards the latter half of surveys and tasks has been shown to reduce the quality of responses or even lead to premature termination of participation, potentially leading to nonresponse bias (15, 16). Factors known to influence this phenomenon include survey length, survey topic, question complexity, and question type, with open ended questions contributing more to exhaustion (16). As the app continues to go through new iterations, it's critical to understand the quality of the responses being received from the app task protocol early on. Users have already expressed dissatisfaction with the length and density of the surveys and questionnaires, highlighting a necessity to create a more streamlined protocol to ensure the best quality of responses. Moreover, the recorded completion time may be affected if the surveys and tasks are not being authentically answered by participants, resulting in a potential under- or overestimation of the test parameter. Future beta-testing in the app should attempt to better understand the influence fatiguability has on response quality and completion time by having participants complete more tasks by bundling tasks across the app. While there is no universal standard for mobile health apps and the time it takes to complete certain protocols, one goal of the application protocol should be to reduce user burden while still collecting sufficient comprehensive data.

With respect to the exit survey interviews, participants were receptive to this mobile application as a future screening tool and support the utility of voice-screening tools in disease diagnosis, screening, and maintenance based on participant opinions. However, a recurrent theme that presented itself from feedback was that there is a need for more explicit instructions and the incorporation of a more better user experience (UX). This ambiguity of task instructions poses a significant challenge to the app's utility, as exemplified by the large, measured percentage of participants that required assistance. Since the app was developed by clinicians and scientists, some of the language used in the surveys, questionnaires, and audio tasks could reflect a higher reading level. Future iterations of the app should investigate the current reading level using existing tools, like the Flesch-Kincaid readability tests, and seek to match the health literacy of the general population (17). The addition of questionnaire to gauge healthy literacy may help better understand the population this app intends to serve. Beyond instruction clarity, there was a call by participants for a more user-friendly interface. The app as it stands is in the process of developing its aesthetic and translating that into the UX. However, we are seeing very early into beta-testing how common comments are on the design of an app and can affect the UX by participants. Once the interface is thoroughly developed, future beta-testing should include more questions from the MARS questionnaire to evaluate UX. Addressing these concerns can further optimize the user experience, potentially improving some of the metric measures.

Audio data has already been shown to serve a potential diagnostic tool in patients with certain disease states that can present with unique vocal changes, including Parkinson's disease, chronic obstructive pulmonary disease (COPD), diabetes, chronic pain, and laryngeal cancer to name a few (18–22). However, without adequate high-quality voice samples, the reliability and accuracy of voice biomarker research may be restricted. The integration of voice-based assessments into digital health tools has the potential to advance early disease detection, continuous disease monitoring, and personalized treatment strategies. For example, an individual using a voice-assessment tool through an app may be identified to have softened consonants, abnormal silences and monotonous speech may point to recommended evaluation for Parkinson's disease (18). Through ongoing refinement, the Bridge2Ai-Voice app attempts to bridge the gap between research and clinical utility.

### Limitations

This study is not without limitations. Firstly, this feasibility study was conducted at a single site. Consequently, this leads to a small sample size (*n* = 47) without geographical diversity, as evidenced by some of the demographic information collected (i.e., primary language, gender). The homogeneity reflected in our demographics greatly limits the generalizability. As the app continues to be modified and early feedback is incorporated, research around the practicality and utility of this app needs to expand to other sites within the consortium to have a more robust, diverse study population to better understand the measured metrics and interview responses as they relate to different subgroups of the populations. As a feasibility pilot, this study did not have a control arm and thus we were unable to test the true efficacy or other measured metrics between groups. Moreover, the determination of whether a task was successfully completed or recorded was subjective as it was made at the decision of the research assistant. While the research assistants are trained on the tasks, a more structured framework with objective benchmarks for what is considered successful vs. unsuccessful in regard to completion and acoustic recording could help with future testing in pinpointing specific faults within the protocol.

## Conclusions

The findings of this pilot feasibility study indicates that the Bridge2AI-Voice smartphone application shows promise as a tool for voice data collection. However, several challenges need to be addressed to enhance its practicality. Refinement of task instructions, interface design, and incorporation of engagement enhancement strategies are crucial for maximizing the app's utility in voice data collection. The smartphone app is need for further adaptation and refinement before large scale voice data collection can be implemented in real-world settings.

## Data Availability

The raw data supporting the conclusions of this article will be made available by the authors, without undue reservation.

## Group members of Bridge2AI-Voice Consortium

University of South Florida, Tampa, FL, US: Yael Bensoussan. Weill Cornell Medicine, New York, NY, USA: Olivier Elemento. Weill Cornell Medicine, New York, NY, USA: Anais Rameau. Weill Cornell Medicine, New York, NY, USA: Alexandros Sigaras. Massachusetts Institute of Technology, Boston, MA, USA: Satrajit Ghosh. Vanderbilt University Medical Center, Nashville, TN, USA: Maria Powell. University of Montreal, Montreal, Quebec, Canada: Vardit Ravitsky. Simon Fraser University, Burnaby, BC, Canada: Jean Christophe Belisle-Pipon. Oregon Health & Science University, Portland, OR, USA: David Dorr. Washington University in St. Louis, St. Louis, MO, USA: Phillip Payne. University of Toronto, Toronto, Ontario, Canada: Alistair Johnson. University of South Florida, Tampa, FL, USA: Ruth Bahr. University of Florida, Gainesville, FL, USA: Donald Bolser. Dalhousie University, Toronto, ON, Canada: Frank Rudzicz. Mount Sinai Hospital, Sinai Health, University of Toronto, Toronto, ON, Canada: Jordan Lerner-Ellis. Boston Children's Hospital, Boston, MA, USA: Kathy Jenkins. University of Central Florida, Orlando, FL, USA: Shaheen Awan. University of South Florida, Tampa, FL, USA: Micah Boyer. Oregon Health & Science University, Portland, OR, USA: William Hersh. Washington University in St. Louis, St. Louis, MO, USA: Andrea Krussel. Oregon Health & Science University, Portland, OR, USA: Steven Bedrick. UT Health, Houston, TX, USA: Toufeeq Ahmed Syed. University of South Florida, Tampa, FL, USA: Jamie Toghranegar. University of South Florida, Tampa, FL, USA: James Anibal. New York, NY, USA: Duncan Sutherland. University of South Florida, Tampa, FL, USA: Enrique Diaz-Ocampo. University of South Florida, Tampa, FL, USA: Elizabeth Silberhoz Boston Children's Hospital, Boston, MA, USA: John Costello. Vanderbilt University Medical Center, Nashville, TN, USA: Alexander Gelbard. Vanderbilt University Medical Center, Nashville, TN, USA: Kimberly Vinson. University of South Florida, Tampa, FL, USA: Tempestt Neal. Mount Sinai Health, Toronto, ON, Canada: Lochana Jayachandran. The Hospital for Sick Children, Toronto, ON, Canada: Evan Ng. Mount Sinai Health, Toronto, ON, Canada: Selina Casalino. University of South Florida, Tampa, FL, USA: Yassmeen Abdel-Aty. University of South Florida, Tampa, FL, USA: Karim Hanna. University of South Florida, Tampa, FL, USA: Theresa Zesiewicz. Florida Atlantic University, Boca Raton, FL, USA: Elijah Moothedan. University of South Florida, Tampa, FL, USA: Emily Evangelista. Vanderbilt University Medical Center, Nashville, TN, USA: Samantha Salvi Cruz. Weill Cornell Medicine, New York, NY, USA: Robin Zhao. University of South Florida, Tampa, FL, USA: Mohamed Ebraheem. University of South Florida, Tampa, FL, USA: Karlee Newberry. University of South Florida, Tampa, FL, USA: Iris De Santiago. University of South Florida, Tampa, FL, USA: Ellie Eiseman. University of South Florida, Tampa, FL, USA: JM Rahman. Boston Children's Hospital, Boston, MA, USA: Stacy Jo. Hospital for Sick Children, Toronto, ON, Canada: Anna Goldenberg.
